# Comparison of Diffusion Kurtosis Imaging and Standard Mono-Exponential Apparent Diffusion Coefficient in Diagnosis of Significant Prostate Cancer—A Correlation with Gleason Score Assessed on Whole-Mount Histopathology Specimens

**DOI:** 10.3390/diagnostics13020173

**Published:** 2023-01-04

**Authors:** Anna Żurowska, Rafał Pęksa, Małgorzata Grzywińska, Damian Panas, Marek Sowa, Katarzyna Skrobisz, Marcin Matuszewski, Edyta Szurowska

**Affiliations:** 1 Second Department of Radiology, Medical University of Gdansk, 80-214 Gdansk, Poland; 2 Department of Pathomorphology, Medical University of Gdansk, 80-214 Gdansk, Poland; 3 Department of Human Physiology, Medical University of Gdansk, 80-214 Gdansk, Poland; 4 Molecular Biology Laboratory, Institute of Animal Reproduction and Food Research Polish Academy of Sciences, 10-748 Olsztyn, Poland; 5 Department of Urology, Medical University of Gdansk, 80-214 Gdansk, Poland; 6 Department of Radiology, Medical University of Gdansk, 80-214 Gdansk, Poland

**Keywords:** prostate cancer, Magnetic Resonance Imaging (MRI), Diffusion Weighted Imaging (DWI), Diffusion Kurtosis Imaging (DKI), Gleason score

## Abstract

Background: The study was undertaken to compare the diagnostic performance of diffusion kurtosis imaging (DKI) with the standard monoexponential (ME) apparent diffusion coefficient (ADC) model in the detection of significant prostate cancer (PCa), using whole-mount histopathology of radical prostatectomy specimens as a reference standard. Methods: 155 patients with prostate cancer had undergone multiparametric magnetic resonance imaging (mpMRI) at 3T before prostatectomy. Quantitative diffusion parameters—the apparent diffusion coefficient corrected for non-Gaussian behavior (D_app_), kurtosis (K), ADC_1200_, and ADC_2000_ were correlated with Gleason score and compared between cancerous and benign tissue and between GS ≤ 3 + 3 and GS ≥ 3 + 4 tumors. Results: The mean values of all diffusion parameters (D_app_, K, ADC_1200_, ADC_2000_) were significantly different both between malignant and benign tissue and between GS ≤ 3 + 3 and GS ≥ 3 + 4 tumors. Although the kurtosis model was better fitted to DWI data, the diagnostic performance in receiver operating characteristic (ROC) analysis of DKI and the standard ADC model in the detection of significant PCa was similar in the peripheral zone (PZ) and in peripheral and transitional zones (TZ) together. In conclusion, our study was not able to demonstrate a clear superiority of the kurtosis model over standard ADC in the diagnosis of significant PCa in PZ and in both zones combined.

## 1. Introduction

Prostate cancer (PCa) is the second most common malignancy in men with an incidence of over 1.4 million and the fifth most common cause of cancer-related mortality with 375,000 deaths worldwide [1].

Due to the heterogeneity of prostate cancer, its clinical course may vary widely, from relatively benign to very aggressive; as a result, the terms non-significant and significant prostate cancer were introduced [2]. 

The utmost challenge of contemporary diagnostics is not only to precisely detect prostate cancer but also to differentiate patients with significant disease, who need radical treatment, from those with non-significant disease, who may only require active surveillance [3].

Multiparametric magnetic resonance imaging (mpMRI) and in particular quantitative parameters of diffusion-weighted imaging (DWI) have been gaining increasing attention as non-invasive biomarkers for the diagnosis and prediction of the degree of malignancy of PCa [4,5,6,7,8,9].

This is due to the ability of DWI to reflect Brownian motions of water molecules in human tissues and to provide indirect information about tumor cellularity and integrity of cellular membranes [10,11]. 

The standard MRI method used for assessing PCa is ADC (Apparent Diffusion Coefficient), calculated using a Monoexponential Model (ME), which assumes a Gaussian (free) diffusion of water molecules and a linear decay of the natural logarithmic diffusion signal intensity as the b-value increases [10,11]. In complex human tissues, this is valid with b values up to 1000 s/mm^2^. With values above 1000 s/mm^2^, an increasing fraction of molecules strike the boundaries of cellular membranes and other molecules; as a result, diffusion deviates from free Gaussian distribution with a loss of linearity of the logarithmic decay plot [10,12,13].

Diffusion Kurtosis Imaging (DKI) is a more complex diffusion model that assumes the non-Gaussian behavior of water molecules at ultrahigh b values. It yields two variables: kurtosis (K)—which describes the extent of deviation of a non-Gaussian from a standard Gaussian diffusion distribution and is measured as a dimensionless quantity— and D_app_—the apparent diffusion coefficient that is corrected for non-Gaussian behavior (expressed in units: 10^−3^ mm^2^/s) [14]. 

DKI potentially adds more microstructural information on PCa, which is histologically heterogeneous with a mixture of higher- and lower-grade components within a single lesion [15]. It is assumed that DKI may result in improved separation of different tissue types [16].

To date, several studies have evaluated the diagnostic performance of DKI rendering inconsistent results [16,17,18,19,20,21,22,23]. The discrepancy in the presented results may be due to the use of different reference standards, various methods of obtaining quantitative diffusion parameters, and small patient numbers in older studies. As a result, the added value of kurtosis in comparison with standard ADC for the characterization of prostate cancer is not clearly established.

The purpose of our study was to compare the diagnostic performance of DKI, including multivariable models combining several diffusion parameters, with the standard monoexponential DWI model in the detection and characterization of PCa on a large population, using whole-mount histopathology of radical prostatectomy specimens as a reference standard.

In contrast to other recent publications, in our study we exclusively used whole-mount histopathology of radical prostatectomy specimens on a large group of patients as reference standard, which allowed the precise correlation of PCa foci and healthy tissue visible on MRI with histopathology and the most accurate assessment of Gleason score in each prostate cancer focus individually.

Moreover, we investigated the diagnostic performance not only of each single diffusion parameter, but also the combination of both parameters D_app_ and K together derived from the kurtosis model in comparison with conventional ADC, as well as whether the combination of all parameters derived from both diffusion models in comparison with ADC alone could be helpful in the diagnosis of clinically significant prostate cancer, which have not previously been studied.

In addition, we compared the diagnostic performance of both models in different tumor locations—in the peripheral zone (PZ) and in the transitional zone (TZ).

## 2. Materials and Methods

### 2.1. Study Population

This retrospective single-institution study included 155 patients with an average age of 66.14 years (range 51–81), mean Prostate Specific Antigen (PSA) level 9.4 ng/mL (range 2.2–37), with prostate cancer who had undergone mpMRI at 3T before prostatectomy. 

### 2.2. Data Acquisition 

All MRI examinations were performed by a 3T MRI scanner (Philips Achieva 3.0 T Tx) with a 32-channel cardiac coil. The DWI protocol was carried out using a single-shot echo-planar imaging sequence in the axial plane, repetition time/echo time (TR/TE) 2000 ms/70 ms, a field of view (FOV) 180 × 250 mm, matrix 80 × 112, slice thickness of 3.5 mm, and gap 0–0.35 mm. DWI protocol consisted of 6 b values (0, 100, 500, 800, 1200, and 2000 s/mm^2^) with five signal averages per b value. Moreover, the prostate mpMRI protocol consisted of T2-weighted imaging in three planes, T1-weighted imaging, and dynamic contrast-enhanced (DCE) imaging.

### 2.3. Data Analysis

DICOM images were transferred from PACS to dedicated software (Intellispace Portal 10, Advanced Diffusion Analysis application), which was used to generate maps of apparent diffusion coefficient (D_app_) and kurtosis (K) according to the kurtosis model equation:

S(b) = S(0) exp (−b × D + b^2^ × D_app_^2^ × K/6)

using all b values, and ADC with standard monoexponential model:

S(b) = S(0) exp (−bADC)

using b values up to 1200 s/mm^2^ and all b values.

**Figure 1:**
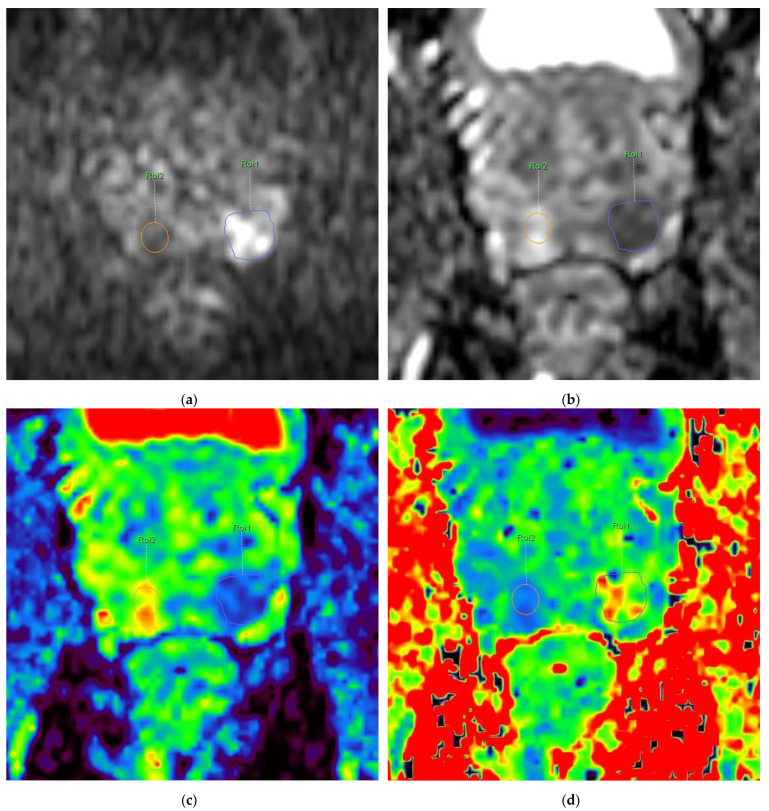

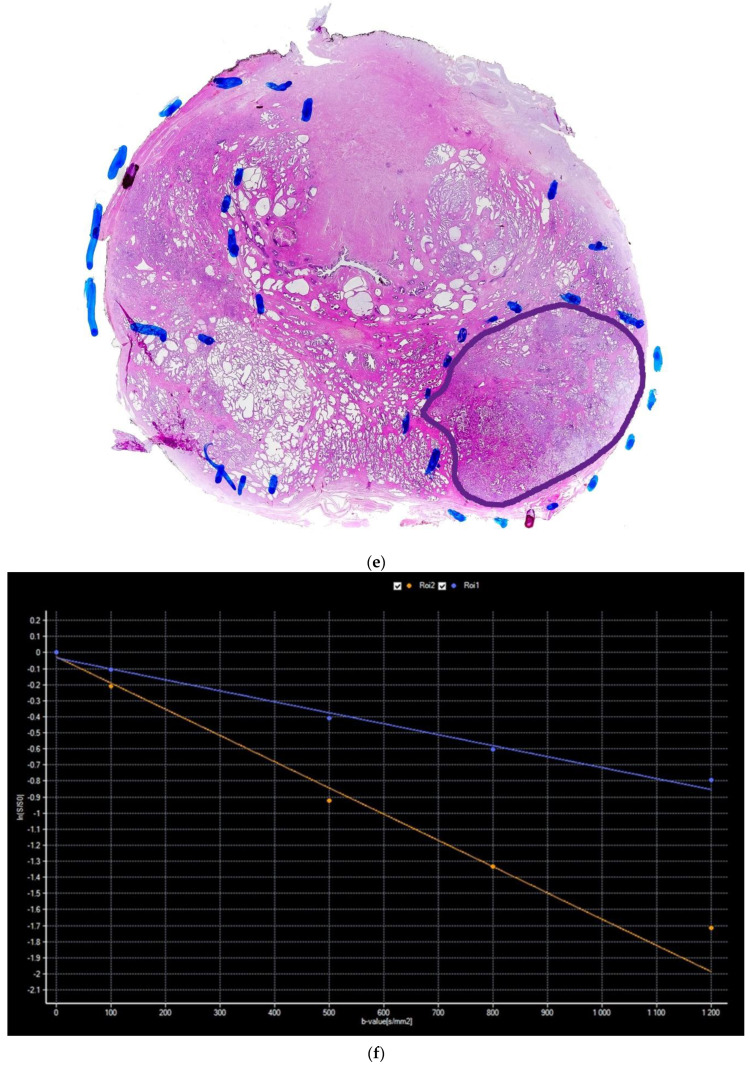

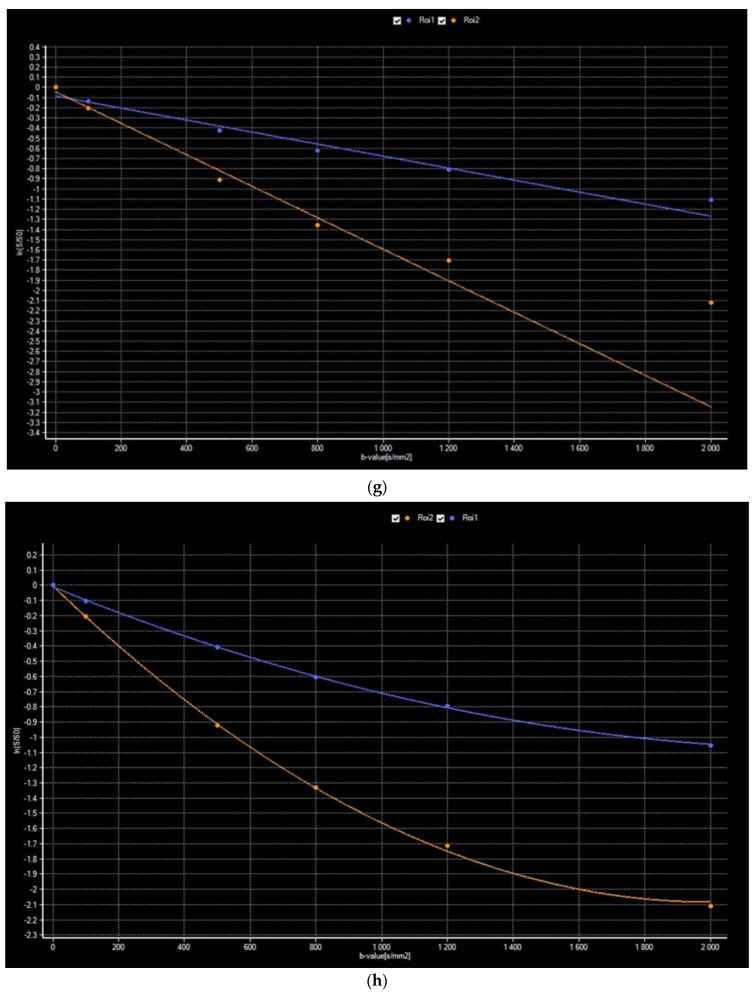
62-year-old patient with PSA level 8 ng/mL and PIRADS 5 lesion located in left PZ, that after radical prostatectomy was assessed as acinar type prostate adenocarcinoma GS 4 + 3. Images of the prostate with GS 4 + 3 lesion in left PZ (ROI 1—circled in blue) and control (ROI 2—circled in orange) in benign prostatic tissue: (**a**) DWI at b-value 2000 s/mm^2^ with high-intensity lesion in left PZ (ROI 1) and control ROI 2 in normal tissue (**b**) ADC map obtained with b-values 0–1200 s/mm^2^ with corresponding low-intensity signal in lesion in left PZ (ROI 1) of 0.68 × 10^−3^ mm^2^/s and control ROI 2 of 1.73 × 10^−3^ mm^2^/s (**c**) Color-coded maps of D_app_ and (**d**) kurtosis obtained with b-values 0–2000 s/mm^2^ show D_app_ of 0.88 × 10^−3^ mm^2^/s and kurtosis of 1.40 in ROI 1 and D_app_ of 2.18 × 10^−3^ mm^2^/s and kurtosis of 0.69 in ROI 2, respectively. (**e**) Whole-mount prostate specimen shows GS 4 + 3 PCa in left PZ (circled with continuous line in violet); additionally, GS 3 + 4 PCa is seen in right PZ (circled with dashed line in blue). DWI signal-intensity decay plots obtained using monoexponential model (**f**,**g**) and kurtosis model (**h**) for different b-values (marked as dots on the graph). Graph in blue corresponds to ROI 1 in GS 4 + 3 lesion; graph in orange corresponds to control ROI 2 in normal peripheral zone (free of cancer): (**f**) Gaussian distribution standard monoexponential model for b-values 0, 100, 500, 800, 1200. Goodness-of-fit for ROI 1—0.96, ROI 2—0.93. (**g**) Gaussian distribution standard monoexponential model for b-values 0, 100, 500, 800, 1200, 2000. Goodness-of-fit for ROI 1—0.92, ROI 2—0.89. (**h**) Non-Gaussian kurtosis model for b-values 0, 100, 500, 800, 1200, 2000. Goodness-of-fit for ROI 1—0.99, ROI 2—0.98. Figure 1a–d,f–g are modified screenshots provided by Advanced Diffusion Analysis application, Intellispace Portal 10.

All exams were reviewed by two radiologists with 4 and 13 years of experience in prostate MRI, who identified by consensus the location and extent of PCa index lesion(s), as well as normal prostatic tissue with correlation to the histopathologic results of the prostatectomy specimens. A PI-RADS (Prostate Imaging–Reporting and Data System) score was assigned according to PI-RADS version 2.1 [24]. The radiologist with 4 years of experience obtained quantitative diffusion parameters by drawing the ROI (region of interest) on each patient’s dominant lesion(s) and benign prostatic tissue on generated maps of ADC_1200_; then the ROIs were automatically copied to ADC_2000_, D, and K maps, respectively, and the quantitative value of each parameter, as well as the goodness-of-fit, were automatically calculated (Figure 1). The ROI in the PCa foci was drawn inside the lesion excluding margins to avoid a partial volume effect.

### 2.4. Histopathology

Whole-mount prostatectomy specimens served as a reference standard. After radical prostatectomy, the resected prostate was inked and fixed in formalin; then whole-mount processing was performed by slicing into 4 mm sections, perpendicular to the urethra. Each specimen was stained with hematoxylin and eosin.

The Gleason score (GS) of tumors according to 4th Edition of WHO Classification of Tumors of the Urinary System and Male Genital Organs was used to assess the specimens by dedicated pathologists [25]. Neoplastic tissue was defined as any grade of prostate cancer and assessed according to Gleason score, and healthy tissue was defined as free of cancer. 

The insignificant prostate cancer was defined as tumor volume of 0.5 cm^3^, Gleason score 6 (3 + 3) (Grade Group 1), and no extraprostatic extension [2]. 

Cancerous lesions were divided into two groups—low-risk/insignificant PCa (lesions with GS ≤ 3 + 3) and intermediate- to high-risk PCa (lesions with GS ≥ 3 + 4). Quantitative DWI parameters and the combination of DKI parameters together (D_app_ plus K) versus standard ME ADC were compared between malignant and benign tissue and between Gleason score GS ≤ 3 + 3 and GS ≥ 3 + 4 tumors.

Moreover, we assessed whether the combination of all parameters derived from both diffusion models in comparison to standard ADC alone could be helpful in the diagnosis of clinically significant prostate cancer.

Statistical analysis was performed using R 4.1.0. To assess the diagnostic utility of selected diffusion parameters, logistic regression with 10 repeats of 10-fold cross-validation as a resampling method was employed. For each model obtained, a receiver operating characteristic (ROC) was constructed. Specificity, sensitivity, accuracy, and area under the curve (AUC) were calculated. To find the optimal cut-off value, the Youden index was applied. Statistical differences between the AUCs of the models were evaluated with the DeLong test. Levels of diffusion parameters between groups were compared with paired *t*-test or Mann–Whitney U-test. In the case of multiple comparisons, the Holm adjustment method was applied. Across the whole study, a 5% significance level was assumed.

## 3. Results

### 3.1. Tumor Characteristics

A total of 178 tumors were studied. 132 (74%) were located in the peripheral zone and 46 (26%) in the transition zone. 15 tumors were assessed as GS 3 + 3 (low-risk/ insignificant); 53 as GS 3 + 4; 76 as GS 4 + 3; 15 as GS 4 + 4; 19 as GS 4 + 5 and GS 5 + 4 (in total 163 intermediate/high risk). All lesions were visible on DWI images and ADC, D_app_, and K maps. According to PIRADS version 2.1 [24], 4 lesions were classified as PIRADS 3, 86 lesions as PIRADS 4, and 88 lesions as PIRADS 5. General characteristics of the study population are listed in Table 1.

### 3.2. Tumor Detection

**Figure 2:**
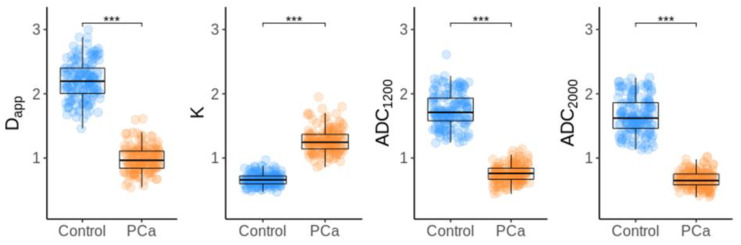
Box-and-whisker plots of DKI parameters and standard ADC in prostate cancer and corresponding normal tissue (*p* < 0.001). *** stands for *p* < 0.001.

All measured MRI parameters calculated by DKI and ME models showed significant differences between tumor and healthy tissues (*p* < 0.001) Figure 2. 

Both ADC_1200_ and ADC_2000_ tumor mean values were significantly lower (0.760 ± 0.140 and 0.663 ± 0.127) than that of mean values obtained from normal prostate tissue (1.743 ± 0.266 and 1.664 ± 0.282). The mean value of D_app_ was significantly lower in tumor (0.982 ± 0.197) compared to healthy tissue (2.205 ± 0.297), and K values were significantly higher in tumor (1.267 ± 0.187) compared to healthy tissue (0.668 ± 0.092). Mean D_app_ values in both tumor and normal prostate tissue were significantly higher than standard ADC values (*p* < 0.001) as shown in Table 2.

All diffusion parameters showed excellent diagnostic performance in the detection of prostate cancer from normal prostatic tissue. 

**Figure 3:**
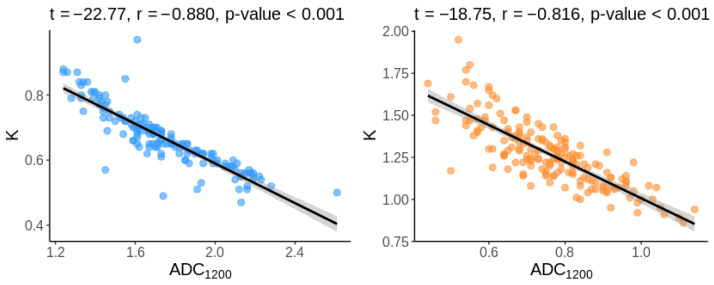
Relationship between ADC_1200_ and K for control tissue (left in blue) and for cancerous tissue (right in orange).

A negative correlation between K and ADC was observed in both PCa (r = −0.816, *p* < 0.001) and benign tissue (r = −0.880 *p* < 0.001) as shown in Figure 3.

### 3.3. Prediction of Clinically Significant PCa

**Figure 4:**
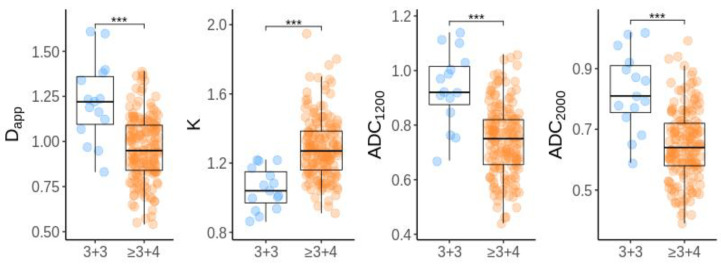
Box-and-whisker plots of mean values for DKI parameters and standard ADC according to Gleason score (GS ≤ 3 + 3 in blue, GS ≥ 3 + 4 in orange) (*p* < 0.001). *** stands for *p* < 0.001.

Mean D_app_, K, ADC_1200_, and ADC_2000_ values were significantly different between low- and higher-grade tumors (*p* < 0.001). In both DKI and ADC parameters, an overlap of values was observed between the low-(GS ≤ 3 + 3) and higher-grade (GS ≥ 3 + 4) tumors (Figure 4). 

Mean D_app_, ADC_1200_, and ADC_2000_ were significantly lower (*p* < 0.001) in GS ≥ 3 + 4 compared to GS 3 + 3 tumors (0.960 ± 0.179 vs. 1.221 ± 0.223 × 10^−3^ s/mm^2^, 0.744 ± 0.129 vs. 0.934 ± 0.137 s/mm^2^ and 0.649 ± 0.117 vs. 0.824 ± 0.129 s/mm^2^, respectively). Mean K values were significantly higher in GS ≥ 3 + 4 compared to GS 3 + 3 tumors: 1.288 ± 0.179 vs. 1.051 ± 0.120 (*p* < 0.001) as shown in Table 3.

**Figure 5:**
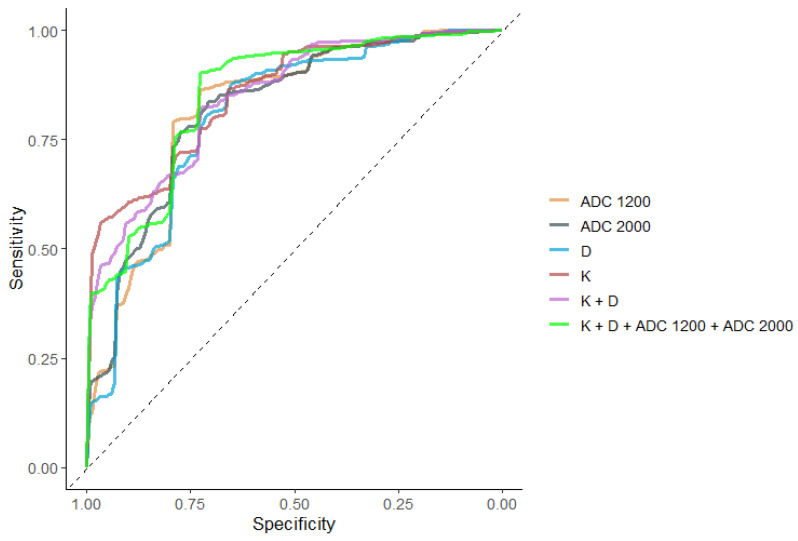
ROC curves of the performance of D_app_, K, ADC_1200_ and ADC_2000_, K + D, and K + D + ADC_1200_ + ADC_2000_ for differentiating GS ≤ 3 + 3 and GS ≥ 3 + 4 tumors located in either PZ or TZ. The models do not differ significantly.

The performance of DKI and standard ADC parameters, as well as the combination of D_app_ and K together and the combination of D_app_ + K + ADC_1200_ + ADC_2000_ together, in differentiating low from intermediate/high prostatic cancers for all tumors, was similar by ROC analysis. Table 4 presents the optimal cut-off values for K, D_app_, ADC_1200_, and ADC_2000_ for the differentiation of low-risk PCa from the intermediate/high-risk group and the respective specificity, sensitivity accuracy, and calculated AUC. AUC curves for the standard ADC and for DKI parameters, as well as for the combination of parameters, were similar as shown in Table 4, Figure 5.

What is more, we assessed the diagnostic performance of both models in discriminating clinically significant PCa (GS ≥ 3 + 4) from clinically insignificant PCa (GS ≤ 3 + 3) depending on the tumor location—in the peripheral zone (PZ) and in the transitional zone (TZ). 

In the peripheral zone, performance of DKI and standard ADC parameters in differentiating clinically insignificant PCa (GS ≤ 3 + 3) from clinically significant PCa (GS ≥ 3 + 4) was similar by ROC analysis.

**Figure 6:**
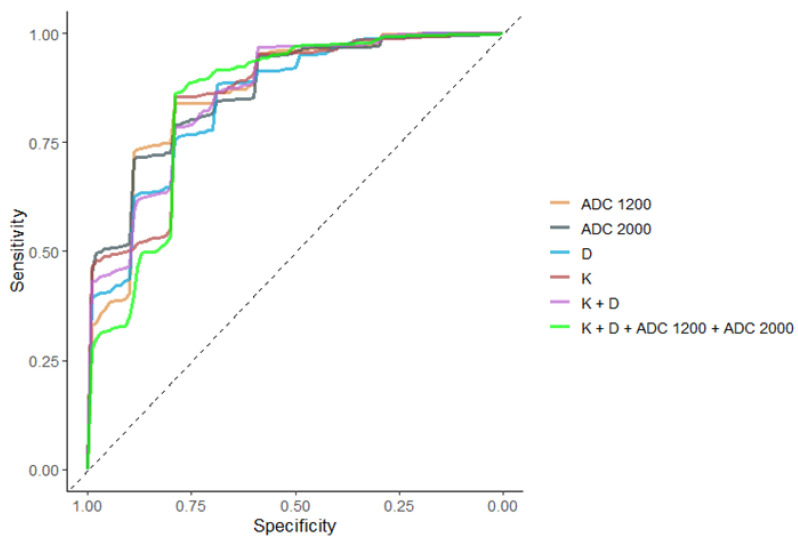
ROC curves of the performance of D_app_, K, ADC_1200_ and ADC_2000_, K + D, and K + D + ADC_1200_ + ADC_2000_ for differentiating GS ≤ 3 + 3 and GS ≥ 3 + 4 tumors in the peripheral zone. The models do not differ significantly.

Table 5 presents the optimal cut-off values for K, D_app_, ADC_1200_, and ADC_2000_ for the differentiation of low-risk PCa from intermediate/high-risk group in PZ and the respective specificity, sensitivity accuracy, and calculated AUC. AUC curves for the standard ADC and for DKI parameters, as well as for the combination of parameters, were similar as shown in Table 5, Figure 6.

In the transitional zone, kurtosis showed statistically significant better diagnostic performance in ROC analysis in comparison with ADC_1200_ (*p* < 0.018) and ADC_2000_ (*p* < 0.019). AUC for kurtosis was 0.849, for ADC_1200_ 0.675, and for ADC_2000_ 0.685, respectively. However, the combination of both DKI parameters together (K + D) did not reveal significantly better diagnostic performance in ROC analysis than ADC alone. AUC for the combination of K + D was 0.811. 

The diagnostic performance of the combination of all parameters derived from both diffusion models (D + K + ADC_1200_ + ADC_2000_) also did not reveal better diagnostic performance in comparison with ADC alone. AUC for (D + K + ADC_1200_ + ADC_2000_) was 0.773.

Nevertheless, it should be noted that the calculations in the transitional zone are very preliminary, due to the very small number of tumors with GS 3 + 3 in TZ (only 5), and this requires further investigation on a larger population. 

**Figure 7:**
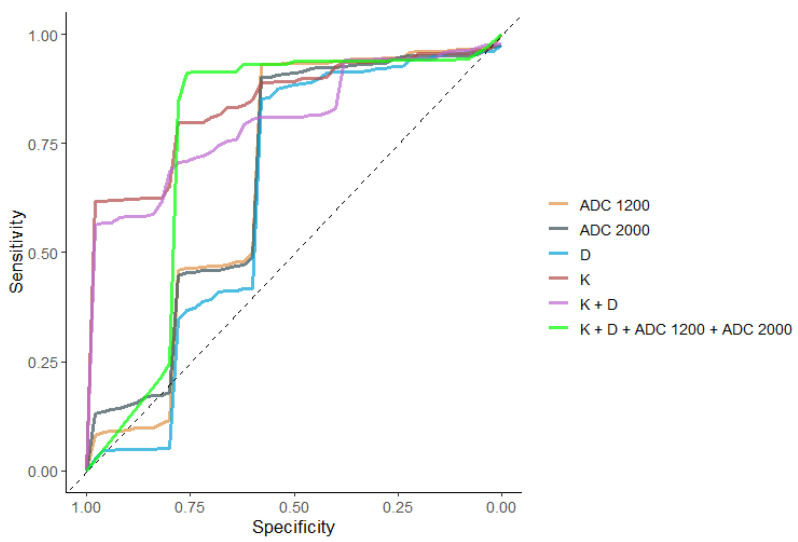
ROC curves of the performance of D_app_, K, ADC_1200_ and ADC_2000_, K + D, and K + D + ADC_1200_ + ADC_2000_ for differentiating GS ≤ 3 + 3 and GS ≥ 3 + 4 tumors in the transitional zone.

Table 6 presents the optimal cut-off values for K, D_app_, ADC_1200_, and ADC_2000_ for the differentiation of low-risk PCa from intermediate/high-risk group in TZ and the respective specificity, sensitivity, accuracy, and calculated AUC. AUC curves were calculated for the standard ADC and for DKI parameters, as well as for the combination of parameters. The AUC curve for kurtosis was statistically significantly higher than AUC for the standard ADC as shown in Table 6, Figure 7.

### 3.4. Goodness-of-Fit to DWI Data

The goodness-of-fit of each model to DWI data was performed by the software (Intellispace Portal 10, Advanced Diffusion Analysis application). The kurtosis model provided the highest fitting performance (mean ± standard deviation): 0.976 ± 0.012 in PCa and 0.975 ± 0.023 in the control region. The standard monoexponential model had slightly lower fitting performance, i.e., 0.954 ± 0.014 and 0.939 ± 0.020 for b-values up to 1200 for PCa and control regions, respectively, and 0.927 ± 0.020 and 0.903 ± 0.026 for b-values up to 2000 in PCa and control regions, respectively.

## 4. Discussion

Multiparametric MRI is gaining broader implementation in the diagnosis of prostate cancer, with an increase in the number of examinations performed. As a result, there is a high demand for a good non-invasive biomarkers in the assessment of prostate cancer aggressiveness. DWI kurtosis is considered to be one of the most promising biomarkers. Although it has been recently investigated in several studies in prostate cancer [17,18,19,20,21,22,23] as well in other pathologies such as gliomas and breast cancer [26,27,28], its role in the diagnosis of prostate cancer has not yet been unequivocally established.

Our study design to compare DKI with standard ADC in the diagnosis of significant prostate cancer, which included only prostatectomy patients, enabled a precise correlation of PCa foci and healthy tissue on MRI with whole-mount histopathology as a reference standard. 

The results of our study confirmed excellent goodness-of-fit of the non-Gaussian Diffusion Kurtosis Model to DWI signal that includes ultra-high b-values, which was better than the goodness-of-fit of the standard monoexponential model to DWI data. 

The means of all measured parameters by DKI and standard ADC were significantly different between prostate cancer foci and normal prostatic tissue. D_app_ and ADC values were significantly lower, and K values were significantly higher in tumors compared to control tissue. Both methods displayed excellent diagnostic performance in the detection of prostate cancer.

Furthermore, we revealed that in both models the mean values of ADC_1200_, ADC_2000_, D_app_, and K were significantly different between low-risk and intermediate/high-risk PCa and might be helpful to predict risk groups. However, an overlap of values is present not only in the ADC assessment but also in the DKI calculations, a finding observed by others in recent publications [16,17,18]. A comparison of DKI and ADC parameters by ROC analysis revealed that in spite of a better fit of DKI to the DWI signal, the diagnostic performance of both methods in the assessment of prostate cancer aggressiveness did not differ significantly in the peripheral zone and in both zones combined.

A slightly higher value of AUC for kurtosis was noted than for ADC in both zones combined, but the difference was not statistically significant. Similar results were revealed by Roethke in the peripheral zone; however, in their research, the DKI parameters were obtained using a separate, longer protocol dedicated to kurtosis, and ADC according to a standard protocol [16]. Furthermore, the diagnostic performance of combination of both parameters D_app_ and K together derived from the kurtosis model in comparison with conventional ADC was similar. Multivariable models investigating the addition of D_app_ and K to standard ADC also did not perform better than ADC alone in the in the diagnosis of clinically significant prostate cancer.

Moreover, in our study, we observed the similar diagnostic performance of ADC_1200_ and ADC_2000_, with no significant differences between them, despite the better fit in the range of b-values up to 1200 s/mm^2^. The demonstration of a statistically significant, strong negative correlation between K and ADC in PCa (r = −0.816, *p* < 0.001) and in benign tissue (r = −0.880 *p* < 0.001), a finding previously noted by Tamada, may to some extent explain the similar diagnostic performance of both models [17,22,23]. 

It is noteworthy that slight differences in the calculated mean values of DWI parameters may exist between publications. 

Values in the ROC analysis for discriminating GS 3 + 3 and GS ≥ 3 + 4 tumors observed in our study were similar to those of Park’s in their PIRADS 5 subgroup analysis and slightly higher than those published by Roethke or Tamada [16,17,18]. AUC in the ROC analysis for discriminating low- and intermediate- to high-grade tumors in our results for K, D_app_, and ADC were as follows: 0.861, 0.806, and 0.822; in the PIRADS 5 subgroup analysis in Park’s paper the results were 0.83, 0.88, and 0.87, respectively [18], whereas in the study of Roethke et al. they were 0.758, 0.769, 0.727 [16], and in the work of Tamada et al. AUC for K was 0.712 and ADC 0.756 [17].

The differences may be the result of the methods used for calculating quantitative parameters and differences in study populations. In the presented study, ROI was drawn in the PCa focus excluding tumor margins on the scan with the lowest ADC, which avoids the partial volume effect, whereas in Park’s and Tamada’s work the whole tumor volume of interest (VOI) was used. Furthermore, the studied cohort consisted mainly of PIRADS 4 and PIRADS 5 lesions reflecting the inclusion of patients only following prostatectomy. 

Additionally, we assessed the diagnostic performance of both models by discriminating clinically significant PCa (GS ≥ 3 + 4) from clinically insignificant PCa (GS ≤ 3 + 3) depending on the tumor location—in the peripheral zone (PZ) and in the transitional zone (TZ). 

In the peripheral zone, performance of DKI and standard ADC parameters in differentiating low from intermediate/high prostatic cancers was similar using ROC analysis.

In the transitional zone, kurtosis showed statistically significant better diagnostic performance in ROC analysis in comparison with ADC_1200_ (*p* < 0.018) and ADC_2000_ (*p* < 0.019). AUC for kurtosis was 0.849, for ADC_1200_ 0.675, and for ADC_2000_ 0.685, respectively. 

However, neither the combination of both DKI parameters together (K + D) nor the combination of all parameters derived from both diffusion models (D + K + ADC_1200_ + ADC_2000_) did not reveal significantly better diagnostic performance in ROC analysis in comparison with ADC alone. 

Nevertheless, it should be noted that the calculations in the transitional zone are very preliminary, due to the very small number of tumors with GS 3 + 3 in TZ in our study population (only 5), and this requires further investigation on larger groups.

In a recent publication, Bingni Zhou et al. [29] revealed that the diagnostic performance of kurtosis in differentiating between prostate cancer located in the transitional zone and stromal hyperplasia was significantly higher than standard ADC. According to our knowledge, no studies were comparing the diagnostic performance of DKI between low-grade and intermediate/high-grade prostate cancer in the transitional zone.

In the presented study, DKI parameters were derived from our standard DWI clinical protocol, which consists of 6 b-values (0–2000 s/mm^2^). ADC maps were obtained with a standard ME model using b-values up to 1200 s/mm^2^ and all b-values. 

Current guidelines [24] recommend using ultra-high b-values in prostate MRI. As a result, DKI parameters can be calculated relatively quickly and easily with dedicated software. However, it requires a larger number of b-values (at least two b-values above 1000 s/mm^2^) for correct calculations, which increases the examination time.

The limitations of our study are its retrospective character and the inclusion of only prostatectomy patients with PCa. The researcher, who obtained quantitative parameters on MRI, was aware of the histopathological findings after proctectomy. While this allowed a precise correlation of PCa and healthy tissue on MRI with histopathology and a precise correlation of MRI foci with GS of whole-mount specimens, it rendered the studied cohort a highly selected group with a relatively small number of subjects with insignificant PCa. In our DWI protocol, slice thickness was 3.5 mm and the gap was 0–0.35 mm. A default parameter for gap parameters is set up to have the proper signal from the acquisition. This value can be changeable in a tolerance of 10% of slice thickness, which should not significantly affect further measurements.

## 5. Conclusions

In conclusion, our study, which used whole-mount prostatectomy as a reference group, was not able to demonstrate a clear superiority of the kurtosis model over standard ADC in diagnosis of significant prostate cancer in the peripheral zone and in the analysis of peripheral and transitional zone tumors combined together. In the very preliminary results on a small number of tumors in TZ, kurtosis showed statistically significant better diagnostic performance in comparison with ADC; however, it requires further research on a larger population. In everyday clinical practice, ADC measurements appear to be simpler, faster, and more accessible with a similar diagnostic performance as the kurtosis model. However, in personalized medicine, additional DKI calculations may potentially add information about tumor heterogeneity, especially in the transitional zone.

## Figures and Tables

**Table 1:** General characteristics of study population.

Parameter	Value
Number of patients	155
Age (years) mean	66.14
range	51–81
Prostate specific antigen level (ng/mL) mean	9.4
range	2.2–37
Number of tumors studied	178
PIRADS 3	4
PIRADS 4	86
PIRADS 5	88
Tumor location N (%) Peripheral Zone	132 (74%)
Transitional Zone	46 (26%)
Gleason score at prostatectomy: score [Grade group]—number of tumors	
3 + 3 [1]	15
3 + 4 [2]	53
4 + 3 [3]	76
4 + 4 [4]	15
4 + 5 and 5 + 4 [5]	19

**Table 2:** DKI diffusion parameters and ADC in cancer and control benign tissue of prostates.

	PCa Mean Value ± SD	Normal Prostatic Tissue Mean Value ± SD	Student *t* Test	*p*-Value
D_app_	0.982 ± 0.197	2.205 ± 0.297	44.7	<0.001
K	1.267 ± 0.187	0.668 ± 0.092	−35.5	<0.001
ADC_1200_	0.760 ± 0.140	1.743 ± 0.266	42.1	<0.001
ADC_2000_	0.663 ± 0.127	1.664 ± 0.282	41.2	<0.001

**Table 3:** Mean values of DKI diffusion parameters and ADC in prostate cancer lesions according to Gleason score.

	GS < 3 + 3	GS ≥ 3 + 4	Mann–Whitney U Test	*p*-Value
D_app_	1.221 ± 0.223	0.960 ± 0.179	2013.5	<0.001
K	1.051 ± 0.120	1.288 ± 0.179	307.5	<0.001
ADC_1200_	0.934 ± 0.137	0.744 ± 0.129	2048	<0.001
ADC_2000_	0.824 ± 0.129	0.649 ± 0.117	2054	<0.001

**Table 4:** Diagnostic performance in differentiating GS ≤ 3 + 3 and GS ≥ 3 + 4 tumors of D_app_, K, ADC_1200_, and ADC_2000_, the combination of D_app_ and K together, the combination of D_app_ + K + ADC_1200_ + ADC_2000_ together, and cut-off values for D, ADC × 10^−3^ mm^2^/s) and K determined by Youden index.

	Cut-Off	Sens.	Spec.	Acc.	AUC (95% CI)	Adjusted *p*-Value				
						D_app_	K	ADC_1200_	ADC_2000_	K + D
D_app_	1.16	0.864	0.667	0.865	0.806 (0.770; 0.849)					
K	1.17	0.730	0.733	0.852	0.861 (0.830; 0.885)	0.344				
ADC_1200_	0.85	0.861	0.733	0.851	0.822 (0.785; 0.863)	1.000	0.916			
ADC_2000_	0.77	0.770	0.787	0.771	0.823 (0.787; 0.861)	1.000	0.917	1.000		
K + D		0.820	0.727	0.812	0.848 (0.818; 0.879)	0.664	1.000	1.000	1.000	
K + D + ADC_1200_ + ADC_2000_		0.902	0.733	0.888	0.856 (0.824; 0.888)	1.000	1.000	1.000	1.000	1.000

**Table 5:** Diagnostic performance in differentiating GS ≤ 3 + 3 and GS ≥ 3 + 4 tumors in the peripheral zone of D_app_, K, ADC_1200_, and ADC_2000_, the combination of D_app_ and K together, the combination of D_app_ + K + ADC_1200_ + ADC_2000_ together, and cut-off values for D, ADC × 10^−3^ mm^2^/s) and K determined by Youden index.

PZ										
	Cut-off	Sens.	Spec.	Acc.	AUC (95% CI)	Adjusted *p*-Value				
						D_app_	K	ADC_1200_	ADC_2000_	K + D
D_app_	1.12	0.851	0.6	0.824	0.853 (0.815; 0.735)					
K	1.10	0.802	0.8	0.802	0.847 (0.804; 0.891)	1.000				
ADC_1200_	0.85	0.932	0.6	0.836	0.873 (0.836; 0.910)	1.000	1.000			
ADC_2000_	0.74	0.905	0.6	0.724	0.875 (0.844; 0.907)	1.000	1.000	1.000		
K + D		0.775	0.80	0.777	0.864 (0.826; 0.901)	1.000	1.000	1.000	1.000	
K + D + ADC_1200_ + ADC_2000_		0.859	0.79	0.854	0.848 (0.802; 0.894)	1.000	1.000	1.000	1.000	1.000

**Table 6:** Diagnostic performance in differentiating GS ≤ 3 + 3 and GS ≥3 + 4 tumors in the transitional zone of D_app_, K, ADC_1200_, and ADC_2000_, combination of D_app_ and K together, combination of D_app_ +K+ ADC_1200_ + ADC_2000_ together, and cut-off values for D, ADC × 10^−3^ mm^2^/s) and K determined by Youden index.

TZ										
	Cut-Off	Sens.	Spec.	Acc.	AUC (95% CI)	Adjusted *p*-Value				
						D_app_	K	ADC_1200_	ADC_2000_	K + D
D_app_	1.16	0.827	0.60	0.802	0.624 (0.523; 0.726)					
K	1.24	0.610	1.00	0.652	0.849 (0.807; 0.892)	<0.001				
ADC_1200_	0.91	0.929	0.60	0.893	0.675 (0.576; 0.774)	0.160	0.018			
ADC_2000_	0.78	0.905	0.60	0.872	0.685 (0.592; 0.777)	0.127	0.019	1.000		
K + D		0.563	1.00	0.611	0.811 (0.764; 0.858)	0.022	0.741	1.000	1.000	
K + D + ADC_1200_ + ADC_2000_		0.912	0.78	0.898	0.773 (0.680; 0.866)	0.387	0.893	0.970	0.971	1.000

## Data Availability

The detailed data presented in this study are available from the corresponding author upon request.

## Abbreviations

DWI—diffusion-weighted imaging; ME—monoexponential model; DKI—Diffusion Kurtosis Imaging; ADC—apparent diffusion coefficient; D_app_—the apparent diffusion coefficient, that is corrected for non-Gaussian behavior; K—kurtosis; PIRADS—Prostate Imaging—Reporting and Data System; mpMRI—multiparametric MRI; PCa—prostate cancer, GS—Gleason score; PSA—prostate-specific antigen; ROI—region of interest; ROC—receiver operating characteristic; AUC—area under the curve.
